# Zero-Shot Water Monitoring from Fixed Drainage Surveillance Imagery Across Day/Night and Rain/No-Rain Conditions: A SAM 3 Benchmark at 25 Urban Sites

**DOI:** 10.3390/s26206403

**Published:** 2026-10-10

**Authors:** Xintong Du, Huan Ding, Sheng Chen, Yuntao Zhu, Xu Wang, Shuang Zhang, Lu Han, Bo Zhang, Chundu Wu

**Affiliations:** 1 School of Environment and Safety Engineering, Jiangsu University, Zhenjiang 212013, China; 2112209003@stmail.ujs.edu.cn (X.D.); zhuytujs@163.com (Y.Z.);; 2 Zhenjiang Drainage Management Office, Zhenjiang 212008, China; 3 School of Agricultural Engineering, Jiangsu University, Zhenjiang 212013, Chinashua.zhang@stmail.ujs.edu.cn (S.Z.)

**Keywords:** urban waterlogging, water-surface segmentation, zero-shot learning, Segment Anything Model, surveillance camera, all-weather monitoring, false-alarm analysis, edge deployment

## Abstract

Urban drainage systems rely on fixed surveillance cameras that operate continuously under daytime, nighttime-infrared, rainy, and non-rainy conditions, yet most vision-based water-monitoring methods are validated only on daytime imagery and require costly pixel-level annotation. This paper investigates whether a promptable segmentation foundation model can monitor water surfaces and waterlogging zero-shot—without any task-specific training or labeling—across the full illumination–weather spectrum of real surveillance operations. We construct a benchmark of 3651 frames from 25 fixed cameras of an urban drainage monitoring network in Zhenjiang, China, spanning four scene categories (river/canal, road ponding, underpass, pump-station apron), seven days in July–September 2026, and all four illumination–weather quadrants, explicitly including non-rainy negative days. Using SAM 3 with the text prompt “water”, we extract per-frame water-area ratio and normalized waterline level, and evaluate (i) quadrant-wise segmentation stability, (ii) rain-event detection, (iii) false-alarm behavior on negative days, and (iv) pixel-level agreement with expert reference masks. Zero-shot segmentation met the scene-specific validity criterion on 96.9% of the 162 diurnal-cycle frames at the canal reference site (100% in both no-rain quadrants, 88.9% in the night-rain quadrant); failures concentrate in heavy-rain-obscured and sediment-turbid dusk frames. On 10 August 2026—a day with two visually verified heavy-rain events—the waterline indicator detected both events (peaks 72.9% and 71.4% vs. a 43–62% control-day band), and the same afternoon event was independently corroborated by severe ponding at road and underpass sites city-wide. A level-only alarm rule produced an 11.8% false-alarm rate on 102 negative-day frames, traced to a camera re-aiming event that shifted the normalized indicator distribution; a compound rule combining area anomaly and rise rate produced no alarms on the 102 examined negative-day frames (vs. 12/102 for the level-only rule) while preserving both event detections—a retrospective, in-sample result that we do not treat as a validated false-alarm rate. We therefore froze the complete pipeline and rules and evaluated them prospectively on 14 additional days (7 days with event-triggered 5-min capture and 7 routine hourly capture days; 1072 frames) that had never been used for threshold or rule design: on the 13 visually verified dry days (784 frames) the frozen compound rule produced only six alarm frames (0.77%), all at illumination-mode switchover transients, and a two-frame persistence variant eliminated all of them (0/13 days; upper 95% confidence bound on the day-level false-alarm rate 20.6%) while detecting both the nighttime rise and the afternoon flood stage of a newly encountered heavy-rain day. Pixel-level verification on an expanded stratified subset of 79 frames from 24 sites with expert reference masks yields a mean IoU of 0.633 (0.652 excluding two failure frames) and near-zero false positives on 15 water-free frames (mean 0.15% of pixels); the reported metrics change by at most 0.003 mIoU under ±5-pixel annotation boundary tolerance. Against two same-protocol baselines—an open-vocabulary detector (YOLOE, mIoU 0.268) and a classic HSV color rule (mIoU 0.453, 68% false-positive area on negative frames)—zero-shot SAM 3 is the only approach that remains accurate in the nighttime-infrared quadrants, and its mIoU varies by less than 0.04 across confidence thresholds 0.15–0.35 at the reported prompt and stays within 0.48–0.66 across four prompt wordings. Reference-mask reliability was independently confirmed by a second annotator (inter-annotator IoU 0.71, Dice 0.80). Inference costs ~46 ms per frame on a consumer laptop GPU (not an edge platform), supporting further evaluation of multi-camera processing. These results establish text-prompted foundation models as a practical, training-free candidate for drainage surveillance across day/night and rain/no-rain conditions—promising for operational evaluation, though not yet operationally validated—and delineate the specific conditions under which they fail.

## 1. Introduction

Rapid urbanization and the increasing frequency of extreme precipitation events have made urban waterlogging a recurring hazard in many cities worldwide [1]. Timely, spatially continuous monitoring of water surfaces—rivers and canals, flooded roads, underpasses, and pump-station forebays—is essential for drainage dispatching, traffic control, and public-safety early warning. Conventional water-level sensors provide accurate point measurements but are costly to deploy and maintain at scale, whereas fixed surveillance cameras already blanket urban drainage infrastructure and offer a dense, low-cost sensing modality that remains largely underexploited [2,3].

Deep-learning approaches to water monitoring have progressed along two lines: water-extent segmentation [4,5,6] and image-based water-level estimation [7,8]. Both lines, however, share three persistent limitations. (i) Annotation cost. Pixel-level labeling of flood imagery is expensive—the FloodNet dataset documentation reports on the order of one hour of annotation per image [9,10]—which discourages large, diverse surveillance datasets. (ii) Daytime bias. Public datasets used in the works reviewed here, including FloodNet [10] (aerial, 2343 images) and the recent low-light street flood dataset behind Aunet [6], cover limited illumination conditions; Aunet’s training set additionally contains almost exclusively flooded imagery, which its authors identify as a source of false positives [6]. Recent waterlogging benchmarks likewise focus on flooded imagery under favorable conditions [11]. (iii) Absence of systematic all-weather evaluation. Field studies have reported notable accuracy drops under rain (e.g., an 11–12% precision drop for object detection in river monitoring [3]), but the evaluations reviewed here do not jointly address day/night and rain/no-rain conditions in a quantitative benchmark for water segmentation from fixed drainage cameras.

The emergence of promptable segmentation foundation models offers a fundamentally different operating point. The Segment Anything Model (SAM) [12] and its successors generalize across domains without task-specific training. Although task-adapted SAM variants have shown promise in flood mapping—with sparse point prompts, fine-tuned SAM attains IoU up to 0.90 [13], and SAM-generated weak labels have enabled low-cost flood delineation [14]—such fine-tuned performance does not by itself establish zero-shot transfer to fixed surveillance imagery. The latest generation, SAM 3 (“Segment Anything with Concepts”) [15], accepts open-vocabulary text prompts and is designed to segment all instances matching a concept—raising the practical question this paper answers: can a text-prompted foundation model replace trained task-specific models for routine, all-weather drainage surveillance, and where exactly does it break?

Answering this question requires data that supervised benchmarks lack: continuous diurnal coverage of the same fixed viewpoints across rainy and non-rainy days, including explicit negatives and operational confounders. We assembled such a benchmark from a production urban drainage monitoring network and conducted a quadrant-wise zero-shot water-segmentation evaluation on fixed surveillance imagery.

Positioning against the closest works. Three recent studies define the state of the art for surveillance-based urban water monitoring, and each differs from the present work in a specific, structural way. NWseg [4] extracts nighttime flood extent from surveillance imagery with a task-trained model; it addresses the night dimension but requires annotated training data and evaluates only on flooded nighttime scenes, without day/night controls or negative days. Aunet [6] monitors low-light waterlogging from surveillance videos with a supervised illumination-invariant network; its authors explicitly attribute residual false positives to the near-absence of non-flooded training imagery—a limitation we address in our evaluation design with five verified negative days and 15 water-free reference frames. The ECCV 2024 waterlogging benchmark [11] provides a challenging annotated testbed and a large–small model co-adapter, but its imagery is dominated by flooded scenes under favorable conditions and all reported results require task-specific training. To the best of our knowledge from the published reports, none of the three evaluates zero-shot foundation models, reports quadrant-wise (day/night × rain/no-rain) performance, evaluates alarm logic on negative days with operational confounders (e.g., camera re-aiming), or compares against open-vocabulary or classical baselines on identical reference masks. Our study is complementary to all three: it asks not “how accurately can a trained model segment floods?” but “how far does an off-the-shelf text-prompted model go in continuous all-weather operation, and where exactly does it break?” Our contributions are:

1. A multi-site day/night × rain/no-rain benchmark (UWS-City). We use 3651 frames from 25 fixed cameras in four scene categories (river/canal, road ponding, underpass, pump-station apron), covering July–September 2026, all four illumination–weather quadrants, two independent heavy-rain-event days, and five non-rainy negative days (Section 3).
2. A zero-shot monitoring pipeline. Text-prompted SAM 3 segmentation followed by water-area and waterline-level indicators and a compound event-detection rule, requiring no training, no annotation, and running at ~46 ms per frame on a consumer GPU (Section 4).
3. A systematic quadrant-wise evaluation with pixel-level verification and baselines. We quantify zero-shot segmentation stability across day/night × rain/no-rain (Section 5.1), detect both heavy-rain events of 10 August 2026 with cross-site corroboration (Section 5.2 and Section 5.5), and show that, in this retrospective in-sample evaluation, a compound alarm rule reduced the observed negative-day alarm frames from 12/102 to 0/102 under a real camera re-aiming event (Section 5.3); we then froze the pipeline and rules and validated them prospectively on 14 additional days never used for threshold or rule design, where the compound rule produced 0.77% alarm frames on 13 visually verified dry days—all at illumination-mode switchover transients—and detected a newly encountered heavy-rain day (Section 5.4). A stratified 79-frame expert-annotated subset spanning 24 sites provides IoU-quantified pixel-level verification (Section 5.7), against which two same-protocol baselines—an open-vocabulary segmenter (YOLOE) and a classical HSV color rule—are benchmarked (Section 5.8), with sensitivity analyses over the model confidence threshold, prompt wording, and alarm-rule parameters (Section 5.9); a failure taxonomy—heavy-rain obscuration, sediment-turbid dusk water, and vegetation-covered water—bounds the method’s operating envelope (Section 5.6 and Section 5.7).

The remainder of this paper is organized as follows. Section 2 reviews related work. Section 3 describes the dataset. Section 4 details the zero-shot pipeline. Section 5 reports experiments, Section 6 discusses limitations and deployment implications, and Section 7 concludes.

## 2. Related Works

### 2.1. Vision-Based Waterlogging and Water-Level Monitoring

Vision-based hydrological monitoring has evolved from handcrafted color/texture features to deep segmentation and regression pipelines. For river scenes, cascaded calibration methods map staff-gauge imagery to metric space via homography before reading the waterline, substantially outperforming calibration-free baselines [7]; SegNet-based water-surface segmentation has reached an RMSE of 0.013–0.066 m against gauge references [8]; and a recent AI camera gauge maintained long-term MAEs of 0.96–2.66 cm across four sites over more than 2.5 years and almost 218,000 images [2]. For urban roads, two strategies dominate: reference-object-based depth grading (e.g., YOLOv4 four-level vehicle-submergence grading at 89.29% accuracy [16]; YOLOv8 five-level grading with mAP@0.5 = 0.96 [17], and flooded-vehicle flood-level detection [18]) and direct water-extent segmentation [4,6,11]. Our work follows the segmentation line but targets routine surveillance rather than disaster imagery, and—unlike gauge-based approaches—estimates normalized rather than metric levels, leaving metric calibration to future work combining the two paradigms.

### 2.2. Nighttime and Adverse-Weather Segmentation

Nighttime semantic segmentation is typically addressed by (i) image enhancement before segmentation, (ii) feature-level illumination disentanglement, or (iii) domain adaptation from day to night. DLA-Net [19] employs progressive daytime-to-nighttime adversarial domain adaptation on Dark Zurich; NightLab [20] proposes a night-aware segmentation framework with light adaptation modules; and illumination-invariant decomposition (e.g., the DisLA module in Aunet [6]) separates lighting components from structural features. These studies establish illumination as a major degradation factor for nighttime imagery and, together with the rain-related drops reported above, motivate evaluating illumination and rainfall jointly; their relative effects in our setting require the controlled analysis that our quadrant-wise design provides. Notably, all of these methods require task-specific training; our study quantifies how far a zero-shot foundation model goes under the same conditions.

### 2.3. Real-Time Segmentation and Edge Deployment

Real-time segmentation on edge devices has been advanced by architectures such as STDC-Seg [21], DDRNet [22], PP-LiteSeg [23], and lightweight SegFormer variants [24]. PP-LiteSeg-T achieves 72.0% mIoU at 273.6 FPS on Cityscapes (GTX 1080Ti, NVIDIA, Santa Clara, CA, USA) [23], while DDRNet-23-slim reaches 77.4% mIoU at 102 FPS (RTX 2080Ti, NVIDIA, Santa Clara, CA, USA) [22]. Deployment studies on Jetson Orin-class devices [25,26] and Rockchip NPUs [27] show that achievable throughput is strongly hardware- and implementation-dependent, with INT8 quantization and vendor runtimes (TensorRT, NVIDIA, Santa Clara, CA, USA; RKNN, Rockchip, Fuzhou, China) providing substantial but workload-specific gains. These works define the supervised efficiency frontier; our zero-shot pipeline is complementary—it requires no per-site training and can serve as an annotation engine for distilling such lightweight models.

### 2.4. Foundation-Model-Assisted Water Analysis

SAM [12] and SAM 2 have been widely adopted as annotation accelerators and zero-shot segmenters. In flood mapping, fine-tuned SAM with point prompts attains IoU ≈ 0.90 [13]; weakly supervised frameworks that adapt SAM to scribble, point, and coarse-mask labels substantially reduce annotation cost while approaching fully supervised segmentation quality [28]; and SAM-generated positive samples have enabled weakly supervised flood delineation in complex scenes [14]. SAM 3 [15] extends promptable segmentation to open-vocabulary text concepts, removing the need for per-image point prompts. Prior hydrological studies, however, evaluated SAM variants on disaster imagery or remote sensing, not on continuous fixed-surveillance operations with day/night and rain/no-rain controls. Our work fills this gap with a quadrant-controlled field benchmark.

## 3. The UWS-City Benchmark

### 3.1. Data Source

Raw imagery originates from the fixed surveillance network of an urban drainage management system in Zhenjiang, China, archived in an on-premises object store organized by site group, camera identifier, and date, with filenames encoding the capture timestamp (hhmmss). Camera identifiers are anonymized throughout this paper following a scene-group-plus-number scheme (Table 1). Cameras capture frames at hourly intervals under routine conditions and switch to 5-min intervals during rainfall events (event-triggered capture), under both daytime color and nighttime-infrared-supplemented imaging. Frames are 1920 × 1080 or 2560 × 1440 JPEG with on-screen date-time overlays.

For this study we curated 3651 frames from 25 camera sites (Table 1), spanning seven days in July–September 2026:

- 10 August 2026 (city-wide heavy-rain day, 3482 frames, all 25 sites). Two visually verified heavy-rain events (approximately 11:15–15:00 and 18:55–22:00 local time) affected the city; independent road and underpass cameras recorded severe ponding at 13:40–13:55, corroborating the afternoon event city-wide.
- 7 July 2026 (second event day, 67 frames, canal reference site RV-001). An evening storm with event-triggered 5-min captures spanning 18:25–23:55. (Nine further frames captured after midnight, whose filenames fall on 8 July 2026, were excluded so that the benchmark covers exactly seven calendar days.)
- Five negative days (102 frames, site RV-001): 18 August, 19 August, 20 August, 26 August, 4 September. No rainfall; used for baseline and false-alarm analysis. Notably, between 26 August and 4 September the reference camera was re-aimed (a pan/tilt maintenance operation changed the field of view), which shifted the normalized indicator distribution on 4 September—a realistic operational confounder, discovered during our pixel-level verification campaign (Section 5.7), that any practical alarm rule must tolerate.

**Figure 1:**
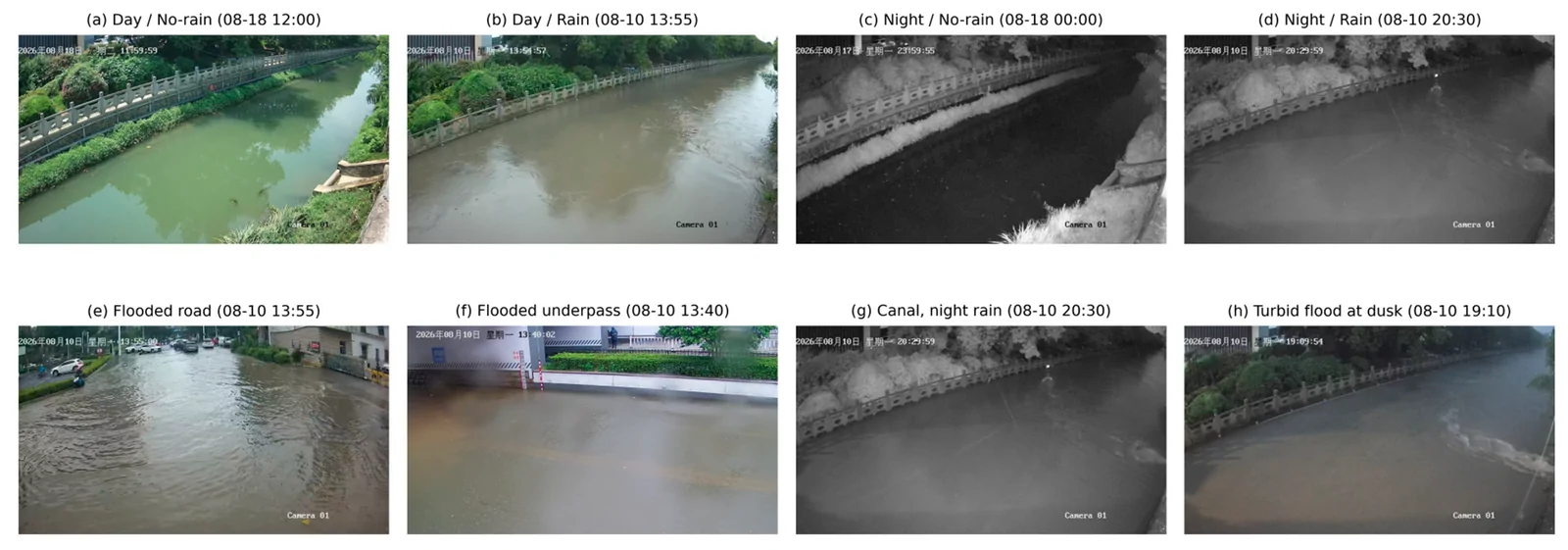
Representative frames of the UWS-City benchmark. (**a**–**d**) The four illumination–weather quadrants at the canal reference site; (**e**–**h**) scene diversity and adverse conditions across sites. The on-screen text in the source frames is the camera’s Chinese date–time overlay (e.g., “2026年08月10日 星期一 13:55:00” reads “13:55:00, Monday, 10 August 2026”); “Camera 01” is a generic camera index.

The canal site RV-001 serves as the reference site for diurnal-cycle and false-alarm analysis because it is the only site with multi-day coverage in the current release; the remaining 24 sites provide same-day cross-sectional diversity on the event day. All four illumination–weather quadrants are populated at the reference site (Table 2). Representative frames are shown in Figure 1.

### 3.2. Curation and Ground-Truth Protocol

Because temporally adjacent frames from fixed cameras are highly redundant, we retained the native capture schedule (hourly routine, 5-min event sequences) rather than uniform sampling, preserving complete event rise/peak/recession sequences. Ground truth at four levels was established as follows. (i) Rainfall windows on 10 August were delimited by visual inspection of frames (visible rain streaks, turbid storm inflow) and confirmed by the network operator as two heavy-rain events; cross-site ponding at 13:40–13:55 provides independent city-wide corroboration. (ii) Negative days were verified frame-by-frame to contain no rainfall and no ponding. (iii) Full-set segmentation quality was screened by visual verification of SAM 3 masks across quadrants, with failure frames logged and categorized (Section 5.6). (iv) Pixel-level reference masks for a stratified 79-frame verification subset (Section 5.7) were manually annotated as polygons following established practice for urban scene benchmarks [29]: the waterline is traced at the base of masonry aprons and vegetated bank strips; fixed structures (gauge platforms, ramps, moored boats) are cut out as holes. The annotation target is inundated-surface extent: objects resting on inundated ground (vehicles, poles, pedestrians) are conservatively counted as water, since the underlying surface is submerged, whereas floating objects that locally displace water (moored boats) are cut out with the fixed structures. This mixed convention—conservative for ground-level occlusion, exclusive where water is displaced—is acknowledged as a design choice and listed among the limitations (Section 6). Wet-but-drained surfaces (damp asphalt without specular standing water) are annotated as water-free, so that the subset tests the practically important wet-vs-ponded distinction. For the fixed-pose reference site, annotation was aided by a level-indexed waterline atlas calibrated on grid-overlaid frames; every mask was verified by overlaid visual inspection in two correction rounds. Of the 25 benchmark sites, one canal site (RV-002) was excluded from pixel-level verification because railings and pipelines occlude the water surface throughout its field of view; the remaining 24 sites are all represented.

### 3.3. Privacy and Security

Frames contain no readable faces or license plates at the working resolution; on-screen overlays show only date, time, and a generic camera index. All camera device identifiers, network topology, and storage endpoints are anonymized or omitted from this paper for infrastructure security; the identifier mapping is held privately by the authors and the network operator. All frames shown in this paper were additionally inspected one by one to confirm the absence of location names, device identifiers, readable plates, and faces.

## 4. Methodology

### 4.1. Zero-Shot Water Segmentation

Given a surveillance frame $I\in R^{H\times W\times 3}$, we obtain a binary water mask $\hat{M}\in \{0,1{\}}^{H\times W}$ directly from SAM 3 [15] (multiplex checkpoint, FP16 quantization, input resized to 644 × 644) using the single English text prompt “water” and a confidence threshold of 0.25. When multiple instances are returned, the final mask is their pixel-wise union:

(1) $$\hat{M}={⋃}_{k}1[s_{k}\geq \tau ]\cdot M_{k},\tau =0.25.$$

**Figure 2:**
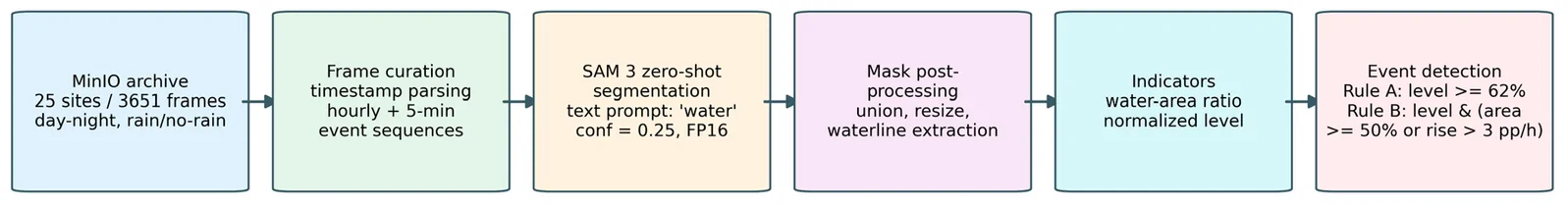
The zero-shot monitoring pipeline: object-store archive → frame curation → SAM 3 text-prompt segmentation → mask post-processing → indicators → event detection (Rule A: level threshold; Rule B: compound level–area–rise rule).

No fine-tuning, prompt engineering beyond the single token, or per-site calibration is applied. The prompt is deliberately minimal: the study measures out-of-the-box transferability, not the ceiling of prompt optimization. Implementation settings: the official SAM 3 multiplex checkpoint [15] with FP16 inference; frames resized to 644 × 644 for inference and masks resampled to native resolution before indicator extraction; the union is taken pointwise over accepted binary instance masks and is defined as the all-zero mask when no instance is accepted. The end-to-end pipeline is illustrated in Figure 2.

### 4.2. Water-Area and Waterline Indicators

From $\hat{M}$ we derive two scalar indicators per frame. The water-area ratio $a=\frac{1}{\mathrm{HW}}\sum \hat{M}$ approximates the visible water extent. The normalized waterline level l locates, for each image column *x* containing water pixels, the topmost water row *y_min_*(*x*), takes the median across columns, and normalizes by image height:

(2) $$l=\left(1-\frac{{\mathrm{median}}_{x}y_{\min}(x)}{H}\right)\times 100\%,$$

computed only when at least 5% of columns contain water; otherwise, the frame is marked as a detection failure. For a fixed camera with a monotonic bank profile, l increases monotonically with true water level, providing a calibration-free relative level indicator [7,8]. Image rows are indexed from the top starting at 0 and normalized by the image height *H*; both indicators are computed in the 0–1 range and reported as percentages (e.g., 0.62 ≡ 62%). The 5%-of-columns validity condition above defines a detection failure at the frame level and is distinct from the 20%-area sanity bound used only for the quadrant statistics at the always-wet reference site (Section 4.4). Because l is defined in image space, a change in camera pose shifts its distribution; Section 5.3 shows how the compound alarm rule absorbs exactly this operational hazard.

### 4.3. Event Detection Rules

An operational alarm must fire on rainfall-driven level rises while ignoring operational disturbances—pump/gate scheduling, and, as our data revealed, camera re-aiming—which can shift indicator distributions without any rainfall. We compare two rules:

- Rule A (level-only): alarm when l ≥ *θ*, with *θ* = 62% set just above the 95th percentile of the negative-day level distribution reported in Section 5.3 (5–95% band 43–62%).
- Rule B (compound): alarm when l ≥ *θ* and (*a* ≥ 50% or the 1-h rise rate of l exceeds 3 pp/h). The rise rate is computed discretely as (l(*t*) − l(*t*^−^))/Δ*t*, where *t*^−^ is the most recent valid frame within 45–75 min before *t*; if no such frame exists (e.g., across day boundaries or long sampling gaps), the rise-rate condition is marked unavailable rather than treated as zero. Rainfall-driven inflow expands the visible water extent and rises fast; operational regulation and slow pose-related offsets do neither.

Within event-triggered 5-min sequences, alarm frames separated by more than 30 min are split into distinct events; under hourly routine sampling, consecutive alarmed hours are treated as one sustained event. Both rules and the threshold were designed from the negative-day data reported in Section 5.3, to which the 4 September day belongs; the alarm evaluation there is therefore retrospective and in-sample, and its zero-alarm outcome should be read as a consistency demonstration rather than an independent prospective validation.

### 4.4. Quadrant Evaluation Protocol

Frames at the reference site are labeled by illumination according to the camera’s actual imaging mode—night from the first infrared frame onward (switchover observed at ≈19:05; nominal boundary 19:00)—and weather (rain = visually verified rainfall windows; no-rain otherwise), yielding four quadrants. Within each quadrant we report the detection success rate (fraction of frames with a valid water mask, area ≥ 20% of the frame as a sanity bound for this always-wet canal scene), and the mean water-area ratio and level (computed over valid frames only; ± values are standard deviations). Pixel-level agreement with expert reference masks (IoU, precision, recall) is evaluated on the stratified 79-frame subset described in Section 5.7, with baseline comparisons in Section 5.8. Runtime is measured on an RTX 4060 Laptop GPU (NVIDIA, Santa Clara, CA, USA; 8 GB), batch 1, FP16.

## 5. Experiments and Results

### 5.1. Quadrant-Wise Segmentation Stability

**Figure 3:**
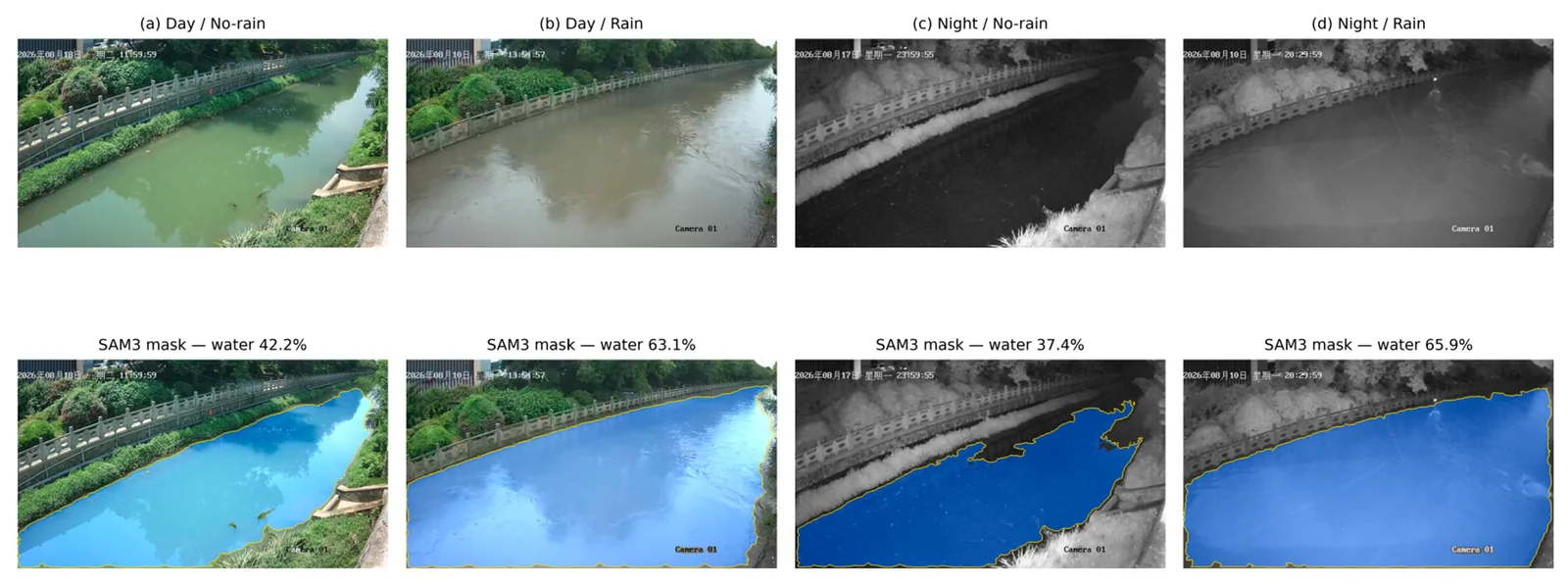
Zero-shot SAM 3 segmentation across the four illumination–weather quadrants (**top**: input; **bottom**: mask overlay). Water-area ratios: 42.2%, 63.1%, 37.4%, 65.9%. On-screen text is the camera’s Chinese date–time overlay (defined in the caption of Figure 1).

Table 2 reports quadrant statistics for the full diurnal cycle of 10 August at the reference site (162 frames). Zero-shot segmentation is highly stable: detection success is 100% in both no-rain quadrants, 97.7% in day-rain, and 88.9% in night-rain, for an overall rate of 96.9% (157/162). Mean water-area ratios are consistent across quadrants (42.0–51.5%), with higher variance under rain reflecting true hydrologic dynamics rather than segmentation noise, as corroborated by the level indicator (Section 5.2). Figure 3 shows representative masks: the model reliably delineates the canal water surface in daytime color and nighttime-infrared imagery alike, including partial walkway ponding at night.

### 5.2. Rain-Event Detection on 10 August 2026

**Figure 4:**
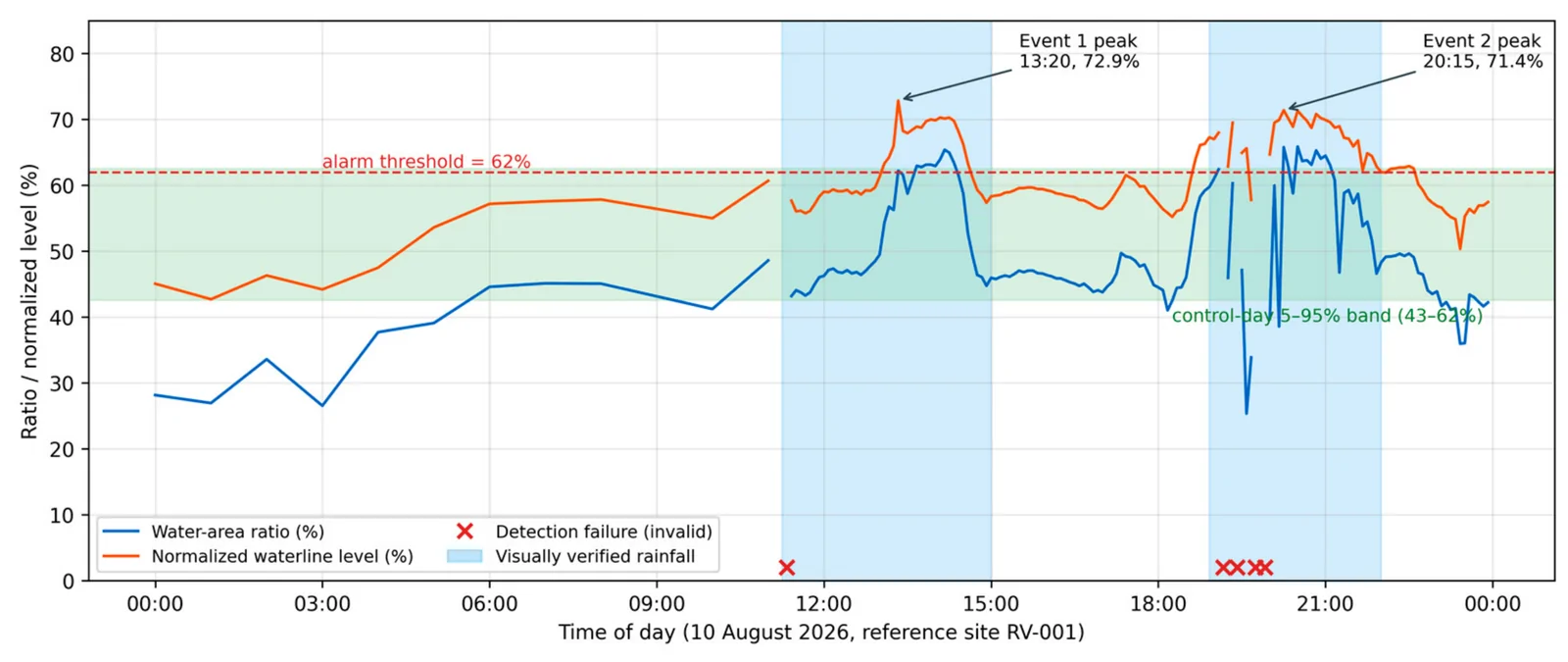
Diurnal hydrograph at the canal reference site on 10 August 2026. Blue: water-area ratio; orange: normalized waterline level; shaded blue: visually verified rainfall windows; green band: 5–95% control-day level range; dashed red: alarm threshold (62%); red crosses: detection-failure frames, whose indicators are treated as missing rather than zero.

Figure 4 shows the diurnal hydrograph at the reference site. Both indicators remain within the control-day band (43–62%) during dry hours and rise sharply during the two verified rainfall windows. Rule A (level ≥ 62%) detects both events: E1 from 13:05 to 14:35 (peak 72.9% at 13:20, area 62.2%) and E2 from 18:40 to 22:35 (peak 71.4% at 20:15, area 65.8%). The timing chain is explicit for both events—for E1: rainfall onset 11:15, alarm onset 13:05, peak (72.9%) 13:20; for E2: rainfall visually identified at the camera site from 18:55, alarm onset 18:40, peak (71.4%) 20:15. The E1 level response lags the visually observed rain onset by ≈110 min, consistent with catchment concentration time into the canal. The E2 alarm precedes the visual rainfall label by 15 min; frame-level inspection clarifies why. The level indicator begins rising at ≈18:15 (55.2%) and accelerates after 18:35 (+2.9–3.4 pp per 5 min), i.e., the hydrological response precedes the alarm. Turbid, sediment-laden storm inflow with churning white water at the outfall structure is already visible at 18:10–18:35, and the stone walkway darkens markedly between 18:30 and 18:55, consistent with rain reaching the camera site around the 18:55 label. We therefore interpret 18:55 as the onset of rain visibility at the camera location—not the onset of the hydrological event—and we annotate the visual rainfall labels as uncertain to roughly ±15 min, since no co-located rain-gauge record is available. Reported against the hydrological rise onset (≈18:15), the alarm trails the event by ≈25 min; reported against the visual rain label, it leads by 15 min. In neither reading is the alarm spurious, and we avoid causal claims beyond what the imagery supports. Rule B preserves both detections (51 alarm frames) while changing the negative-day behavior fundamentally (Section 5.3).

**Figure 5:**
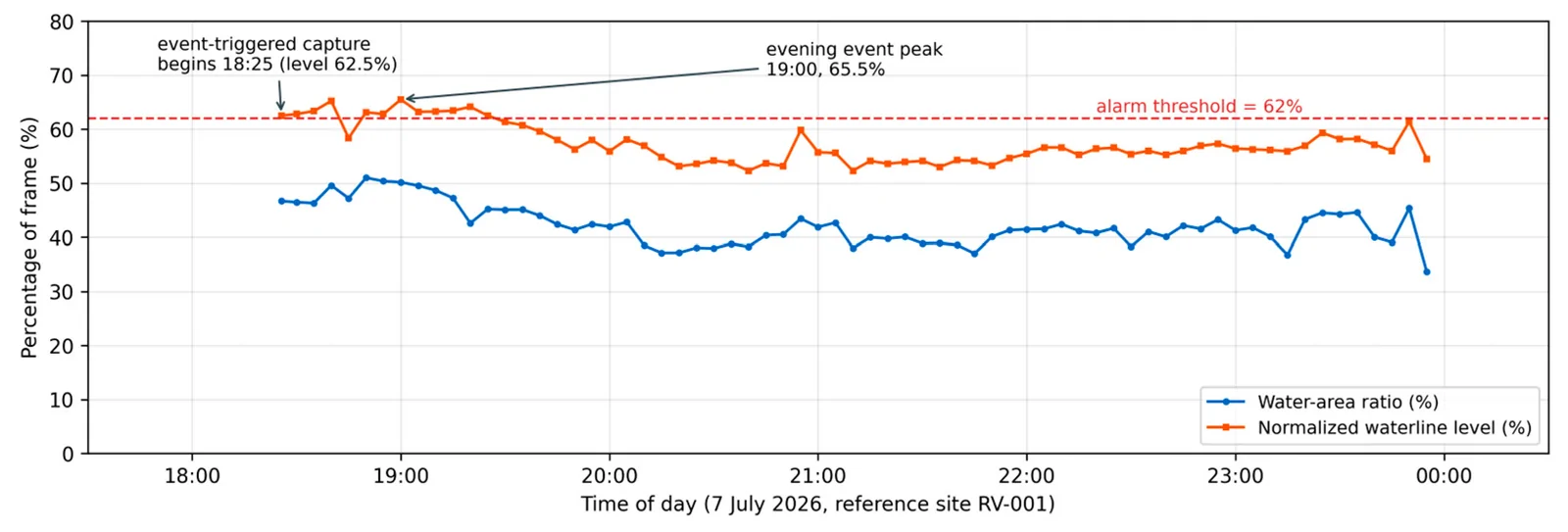
Second event day 7 July 2026 at the reference site: the level stands at 62.5% when event-triggered capture begins (18:25) and rises to the evening storm peak (65.5% at 19:00); dashed red: alarm threshold (62%).

A second event day, 7 July 2026—held out from threshold and rule design—shows the same signature (Figure 5): event-triggered 5-min captures began at 18:25 with the level already at 62.5% and rising during the evening storm, peaking at 65.5% at 19:00. Rule A fired on 12 frames (18:25–19:25); Rule B confirmed the event through its rise-rate condition (≈5 pp/h during 18:25–19:00).

### 5.3. False-Alarm Analysis on Negative Days

Table 3 reports alarm behavior on the 102 negative-day frames. Rule A fires 12 times (11.8% FAR)—all on 4 September. Frame inspection reveals the cause: the reference camera was re-aimed between 26 August and 4 September, and on 4 September it observes a narrower channel reach in which the visible water occupies a systematically larger fraction of the frame, lifting the normalized level indicator to elevated values throughout the day—12 of 22 frames at or above 62%, max 62.9%—with no rainfall (verified visually: dry banks, clear water, sunny conditions; the new pose is unchanged across all 22 frames). Notably, a level-only reading of this day would mimic a slow operational regulation event; only inspection of the imagery itself distinguishes the two, which we did systematically during the pixel-level verification campaign of Section 5.7. Rule B fires zero times (0/102 frames): the pose shift produced a persistent offset in the sampled post-change frames—the camera movement itself was not continuously observed—but it neither expands the water-area ratio beyond 50% nor produces a fast rise, so the compound condition stays silent. Because the compound rule was formulated after inspecting this day (Section 4.3), the 0/102 outcome is a retrospective in-sample result: it demonstrates that a physically motivated compound condition can absorb this confounder, not an independently validated false-alarm rate. This experiment demonstrates two points: (i) explicit negative days are indispensable for evaluating monitoring systems—accuracy on event frames alone would have hidden the confounder entirely; and (ii) a physically motivated compound rule, not a better segmentation model, is what removes this failure mode. We note that camera pose drift is a routine hazard of production surveillance networks (maintenance, vibration, patrol presets), and pose-normalized indicators are an important direction for hardening (Section 6).

### 5.4. Prospective Validation on Previously Unused Days

The zero-alarm outcome of Section 5.3 is retrospective and in-sample—the rule was formulated after inspecting the 4 September confounder—so it demonstrates that a physically motivated compound condition can absorb this failure mode, not an independently validated false-alarm rate. To obtain an out-of-sample estimate, we queried the network archive after the benchmark was finalized and selected 14 additional days at the reference site that had never been used for threshold or rule design: seven days with event-triggered 5-min capture (11/12/13/14 August 2026, 31 August, 1 September, 17 September) and seven routine hourly capture days (24/25/27 August 2026, 8/10/13/15 September), totaling 1072 frames. The pipeline (SAM 3, prompt “water”, confidence 0.25) and both alarm rules were applied exactly as frozen in Section 4.1, Section 4.2 and Section 4.3, without any re-tuning. Rainfall status was verified by frame inspection: every alarm frame and a day-covering sample of routine frames was examined for rain streaks, turbid storm inflow, and ponding.

Dry-day behavior. Thirteen of the fourteen days were verified dry. Rule A (level ≥ 62%) alarmed on 112 of the 784 dry-day frames (14.3%), on 6 of 13 days—almost entirely on September days, where the re-aimed camera observes a narrower channel reach and the normalized level sits systematically higher (cf. Section 5.3). Rule B alarmed on only 6 of 784 frames (0.77%), on 5 of 13 days, and frame inspection shows all six are illumination transients rather than rainfall: four are the first daylight frame after the dawn infrared-to-color switchover (06:00, hourly days), one is a nighttime infrared segmentation drift (13 September, 21:00), and one occurs on 31 August during unstable night infrared imaging with moving foreground vegetation (03:50). No rainfall, turbid inflow, or ponding is visible on any of the six frames or their neighbors.

**Figure 6:**
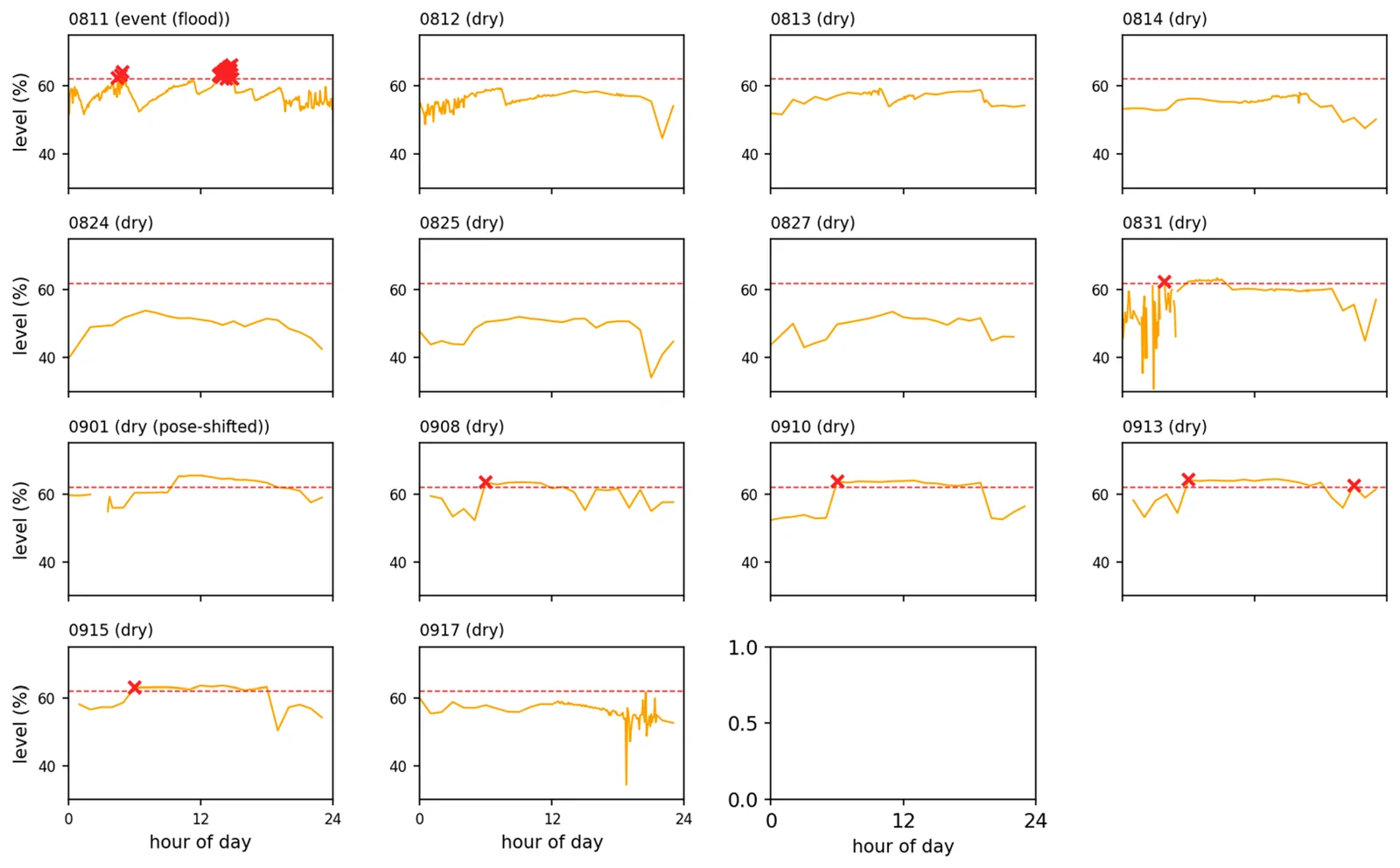
Prospective validation on 14 days never used for threshold or rule design. Normalized waterline level per day (orange), alarm threshold 62% (red dashed). For 11 August: verified flood event, detected. All other days verified dry; the only Rule B alarm frames (red-crossed) are single-frame illumination-switchover transients, eliminated by two-frame persistence.

Event-day behavior. On 11 August 2026—a day with flood-stage turbid water already present from the early morning hours—the frozen Rule B detected both the nighttime rise (04:30–04:55, three consecutive frames) and the afternoon flood stage (13:40–14:55, 16 consecutive frames), both verified against the imagery (Figure 6). Rule A fired on 24 frames of the same day.

A minimal post hoc hardening. Because all six prospective false alarms were single-frame transients at mode switchover, we evaluated the simplest possible remedy, decided only after inspecting these failures: require the alarm condition to hold on at least two consecutive frames (at 5-min sampling, a 10-min persistence; at hourly sampling, two adjacent hours). This variant removes all six false-alarm frames (0/784; 0/13 days; Clopper–Pearson upper 95% bound of the day-level false-alarm rate 20.6%) while preserving both 11 August detections (runs of 3 and 16 frames). Across the 45-point parameter grid of Section 5.9, the persistence variant keeps the original-negative-day alarms at 0/102 and the 11 August detections at all settings. We emphasize that this variant is post hoc with respect to the 14 new days; we report it as a recommended default rather than a validated result, and a fully prospective re-validation of the hardened rule is identified as future work.

### 5.5. Multi-Site Consistency on the Event Day

**Figure 7:**
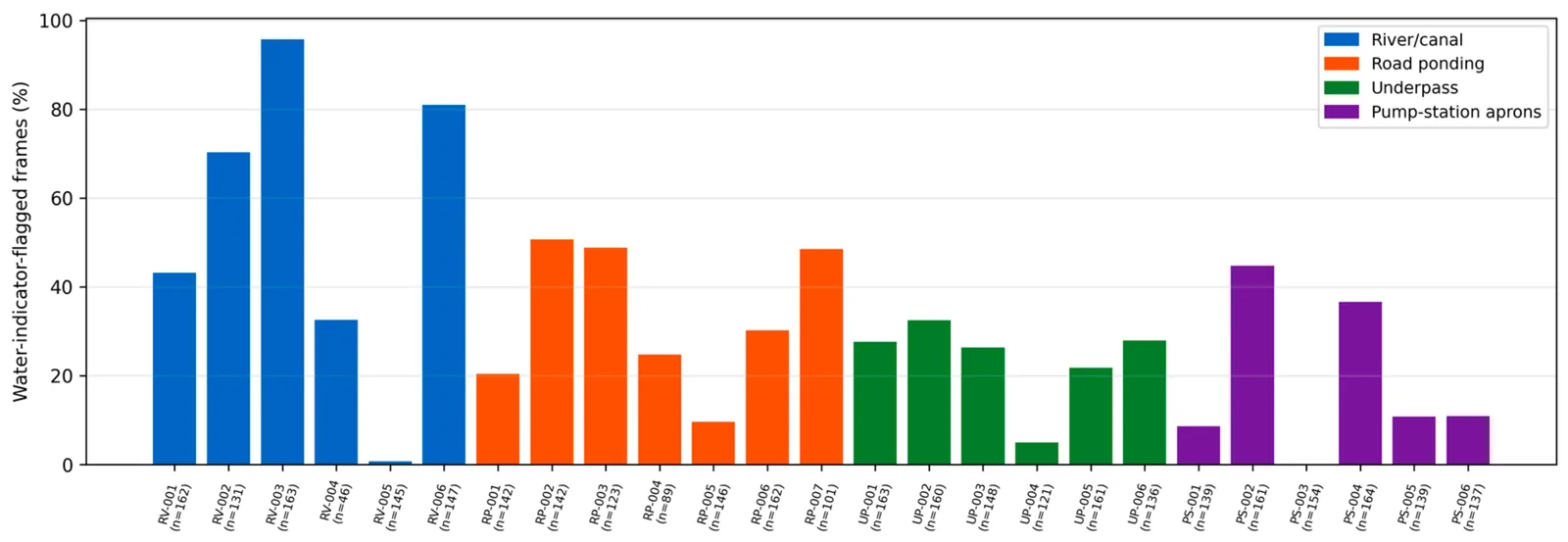
Per-site water-indicator flag ratio on 10 August 2026 across 25 anonymized sites (per-site frame counts beneath the labels) and four scene categories.

Figure 7 summarizes, for all 25 sites on 10 August, the fraction of frames flagged by the scene-appropriate water-indicator rule (level threshold for canal sites; water-area threshold for road, underpass, and pump-station sites), with category ratios pooled over all frames of the category’s sites. These ratios reflect how long each scene type retains visible water and are not comparable across categories as accuracy measures. Rain-flagged ratios differ systematically by scene category—river/canal 57.1%, road ponding 32.6%, underpass 24.2%, pump-station apron 19.5%—reflecting how long each scene type retains visible water after rainfall rather than detection inconsistency: canals integrate upstream inflow for hours, whereas road ponding drains within tens of minutes. Critically, the afternoon event appears synchronously across independent sites: road and underpass cameras recorded their maximum ponding (water-area ratios up to 75.5% and 64.1%) at 13:40–13:55, within 35 min of the canal level peak (13:20), providing city-wide cross-validation that the indicators track the same physical event. We note that this synchronicity is non-trivial: camera spacing in the network ranges from a few hundred meters to more than 10 km with non-negligible elevation differences between sites, rainfall arrival is time-ordered across sites as the rain cell advects with the cloud field, and catchment concentration time adds further offset between rainfall and visible ponding—so small timing differences between sites (minutes to tens of minutes) are the expected operational behavior, and an alignment of independent site maxima within 35 min is accordingly read as same-event corroboration rather than coincidence.

### 5.6. Failure Case Analysis

**Figure 8:**
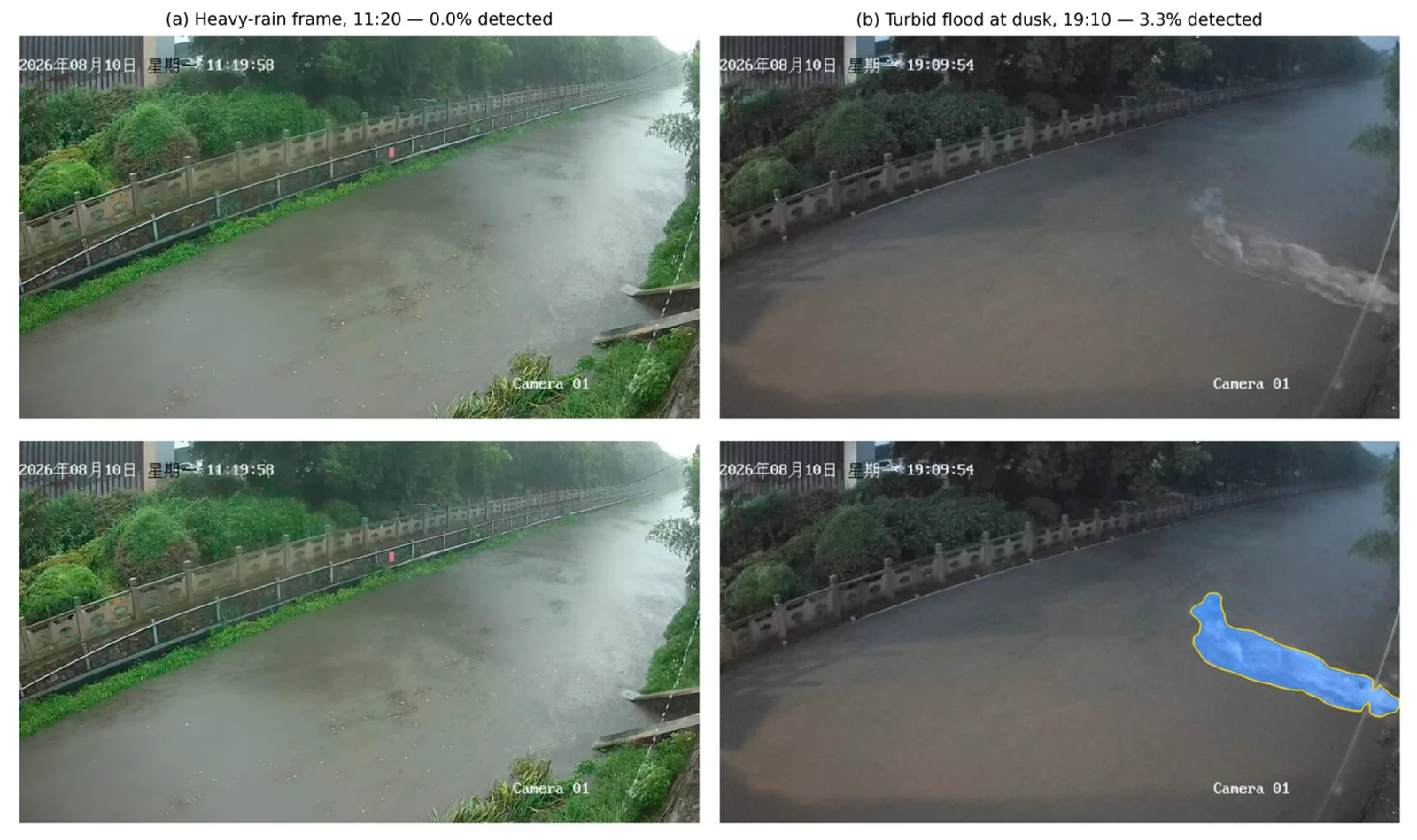
Failure cases of zero-shot segmentation. (**a**) Heavy-rain-obscured frame (11:20, 0.0% detected); (**b**) sediment-turbid floodwater at dusk (19:10, 3.3% detected). On-screen text is the camera’s Chinese date–time overlay (defined in the caption of Figure 1).

All five failure frames (area < 20%) at the reference site—11:20 (0.0%), 19:10 (3.3%), 19:25 (2.1%), 19:45 (15.3%), and 19:55 (17.5% area detected); the first two are included in the 79-frame verification subset—fall into two categories (Figure 8). (i) Heavy-rain obscuration: at 11:20, dense rain streaks and a gray, foam-streaked water surface eliminate the visual signature of “water”; the model returns an empty mask (0.0%). (ii) Sediment-turbid dusk water: between 19:10 and 19:55, sediment-laden brown floodwater under fading light is segmented only partially (2.2–17.5%), recovering as infrared imaging stabilizes. Both categories are transient (single frames to tens of minutes) and concentrate in the night-rain quadrant, explaining its 88.9% success rate. A third, site-specific failure mode—vegetation-covered water (dense duckweed read as land)—emerged from the pixel-level verification at a second canal site (Section 5.7). Together these cases characterize the observed failure modes under the sampled conditions and indicate where task-specific fine-tuning or multi-frame temporal fusion should be invested. Quantitatively, the level indicator’s sensitivity to small camera-pose perturbations—which can themselves induce segmentation failures of exactly these types near the validity boundary—is bounded in Section 5.9 (c).

### 5.7. Pixel-Level Verification Against Expert Reference Masks

**Figure 9:**
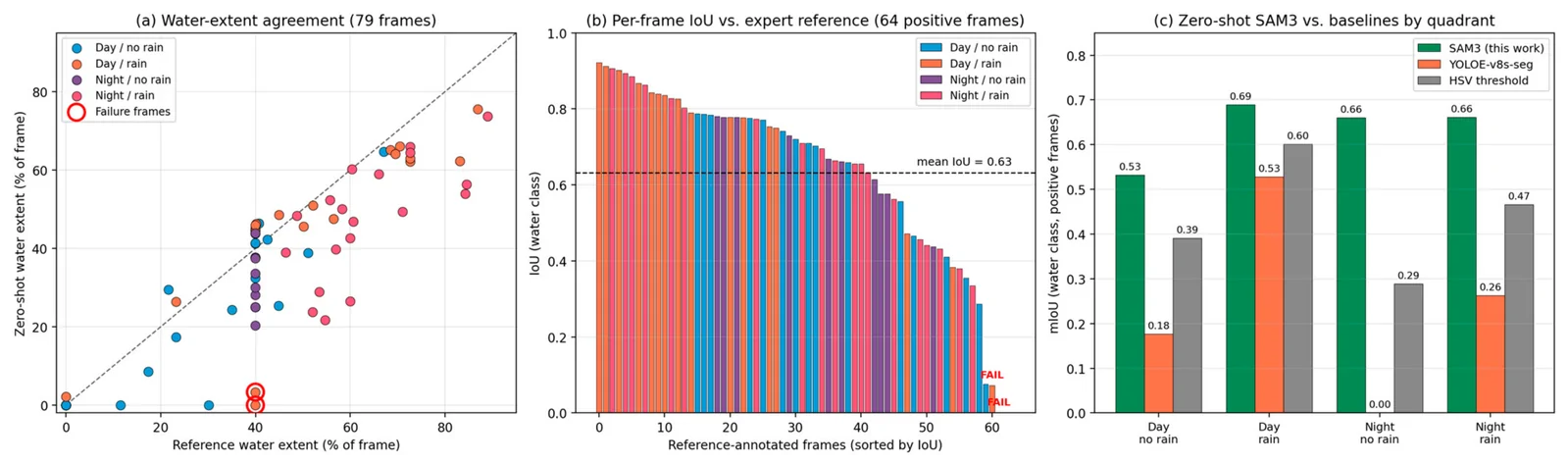
Pixel-level verification on the 79-frame reference subset (24 sites). (**a**) Frame-level water extent: zero-shot vs. expert reference (dashed: identity; red circles: the two failure frames of Section 5.6; points at the origin: water-free negatives). (**b**) Per-frame water-class IoU on the 64 positive frames, sorted, colored by illumination–weather quadrant; dashed line: mean IoU = 0.63. (**c**) Per-quadrant mIoU of zero-shot SAM 3 against the YOLOE and HSV baselines of Section 5.8.

The quadrant statistics above establish detection stability but not pixel-level accuracy. To quantify the latter without compromising the zero-shot study design, we annotated a stratified verification subset after all primary analyses were frozen: 79 frames from 24 sites, comprising 33 reference-site frames spanning the full 10 August diurnal cycle (including both failure frames of Section 5.6), the 7 July event peak, five negative days, and 46 cross-site frames covering every other eligible site at its event-day peak plus a matched pre-event morning reference (08:00, before the day’s first rainfall). Fifteen frames are verified, water-free negatives (wet-but-drained surfaces only). One canal site (RV-002) is excluded because its water surface is occluded by railings and pipelines throughout the field of view. Reference masks follow the polygon protocol of Section 3.2 (iv). Zero-shot masks were recomputed on this subset with the frozen pipeline (Section 4.1); results are reported in Table 4 and Figure 9.

Four findings emerge. (i) Errors stem from boundary ambiguity and appearance-related omissions rather than gross failure. A 20-pixel boundary band around the reference waterline contains only 18.1% of missed pixels, so errors are not concentrated at the boundary alone; the remaining gap reflects a systematic conservative bias along wet bank aprons and an under-coverage of dark infrared water at night (night/no-rain recall 0.704 at precision 0.925). Precision exceeds 0.92 in both night quadrants. Across the 15 water-free negative frames the false-positive pixel rate averages 0.15%: fourteen frames are exactly zero, and the single exception (2.2% of pixels on a wet pump-station apron during rainfall at site PS-003) corresponds to a small specular patch that the annotator judged as film-wet rather than ponded—i.e., residual ambiguity of the wet-vs-ponded boundary, not a gross false alarm. This directly evidences the negative-day specificity underlying Section 5.3. (ii) Performance varies across scene categories and surface states. Road/underpass flooding frames, where ponding has crisp visual extent, score up to 0.91 IoU even at night; the lowest scores come from a canal site whose water surface was densely covered by duckweed (IoU 0.076—the model reads the vegetation mat as land, a third failure mode beyond Section 5.6) and from a boat-occupied reach (0.411). The expanded subset also exposes a systematic weakness we report explicitly: on morning reference frames carrying only thin water films or damp surfaces (e.g., RP-001 and UP-001 at 08:00, before the day’s first rainfall), the model behaves conservatively and returns empty or partial masks where the annotator marked shallow specular sheets, which depresses the day/no-rain quadrant (IoU 0.533, precision 0.673). (iii) Water remains detectable after the pose change. The 4 September reference frame captured after the camera re-aiming still scores IoU 0.556 with zero gross errors—water remained detectable in this examined post-change frame; what shifted is the normalized indicator mapping, which is exactly what the compound alarm rule absorbs (Section 5.3). (iv) Extent estimates remain quantitative. Frame-level water-extent estimates track the reference with Pearson r = 0.783 (Spearman ρ = 0.792, MAE 11.4 percentage points) across the full 0–89% extent range.

Two properties of this verification subset deserve emphasis: the 79 frames are dominated by harder cross-site scenes—thin morning water films, partially occluded views, vegetation-covered water—so the mIoU of 0.633 should not be read as best-case performance; the stratified subset covers selected operational conditions but does not estimate their prevalence in the full archive; and the reference masks remain expert polygons, not pixel-perfect labels, whose boundary convention contributes a few points of irreducible IoU uncertainty. The quadrant-level asymmetries (night precision ≫ recall) and the near-zero negative-frame false-positive rate are robust to this tolerance: an annotation-tolerance analysis that perturbs the reference masks by ±1–5 pixels moves the reported mIoU by at most 0.003 (0.632–0.633), and the perturbed masks retain IoU ≥ 0.95 against the originals (Section 5.9 (d)). Human agreement confirms the same picture: a second independent annotator re-labeled all 79 frames under the identical protocol and agrees with the reference at mean IoU 0.706 and Dice 0.800 on the 64 water-positive frames, with disagreement confined to the thin-film regime (Section 5.9 (e)).

### 5.8. Baseline Comparison

To situate zero-shot SAM 3 within the available alternatives, we evaluated two same-protocol baselines on the identical 79-frame reference set (Table 5, Figure 9c). (i) YOLOE-v8s-seg [30], a state-of-the-art open-vocabulary detection-and-segmentation model, prompted with the same text “water” at the same confidence threshold (0.25)—this isolates the contribution of SAM 3′s segmentation backbone and training data from open-vocabulary prompting itself. (ii) An HSV color-threshold rule (saturation ≤ 60, value within [40, 200], 5 × 5 morphological opening), representative of the classical color-prior pipeline; its thresholds were fixed a priori, before any evaluation on the reference set.

The three methods fail in instructively different ways. YOLOE is conservative: it emits essentially no false positives (0.00% on negative frames) but misses most water (recall 0.302), and it obtains zero mean IoU in the night/no-rain quadrant and only low mean IoU under night rain (Figure 9c), consistent with its RGB-centric pretraining. The HSV rule shows the opposite pathology: acceptable recall (0.730) bought at the price of catastrophic over-detection—on water-free frames it marks a mean of 68% of all pixels as water (max 90%), because wet asphalt, shadows, and infrared grayscale all satisfy the low-saturation prior; it is therefore unsuitable for alarm logic in this setting. Zero-shot SAM 3 is the only method that is simultaneously accurate (mIoU 0.633), specific (0.15% mean false-positive area), and stable across all four illumination–weather quadrants (per-quadrant mIoU 0.53–0.69, Figure 9c). We stress that neither baseline is the strongest member of its family—YOLOE has larger variants, and color rules can be tuned per site—but both represent the training-free operating points available to a practitioner; closing the gap with supervised per-site training is precisely the cost our zero-shot pipeline avoids.

### 5.9. Sensitivity Analyses

We assess how robust the reported results are to the methodological choices that could most plausibly drive them: the SAM 3 confidence threshold and prompt wording, the alarm-rule parameters, and small camera-pose perturbations; and we bound the effect of reference-mask boundary ambiguity.

**Figure 10:**
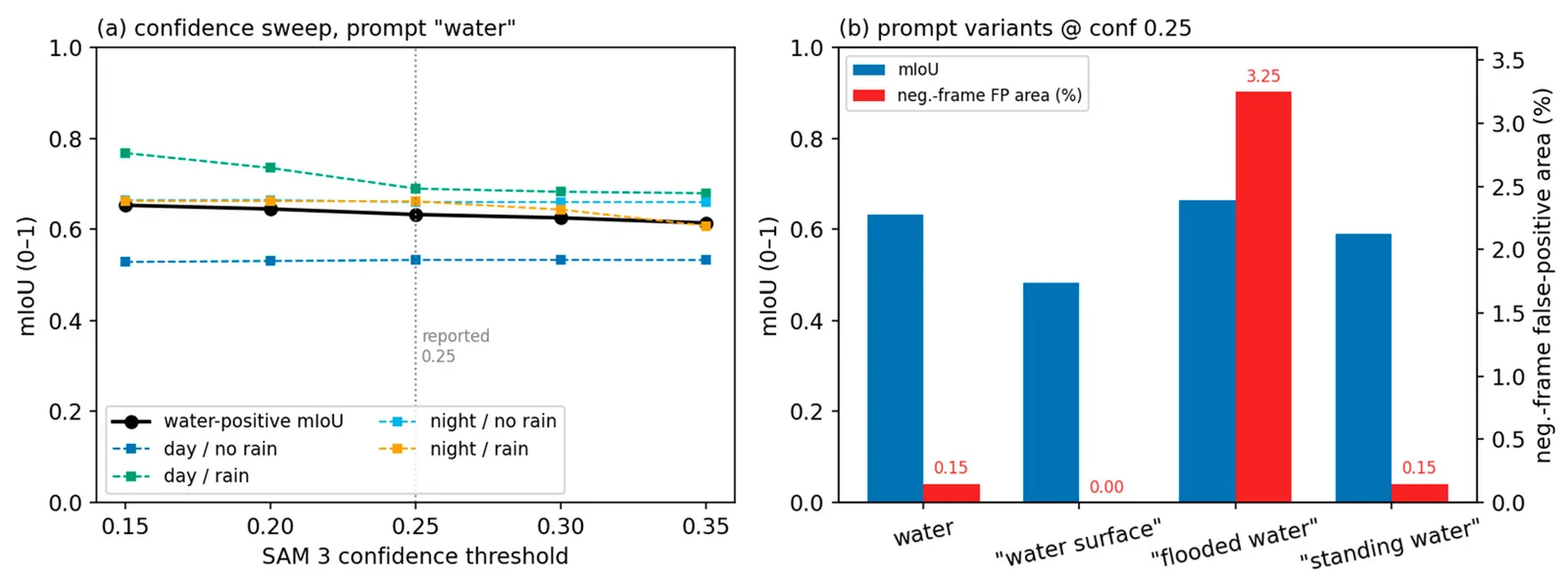
Sensitivity of pixel-level verification to model settings. (**a**) Water-positive mIoU and per-quadrant mIoU as a function of the SAM 3 confidence threshold; the reported operating point 0.25 lies on a flat region. (**b**) mIoU (bars) and negative-frame false-positive area (red markers) for four prompt wordings at confidence 0.25; “flooded water” trades a small IoU gain for a 22-fold specificity loss.

**Figure 11:**
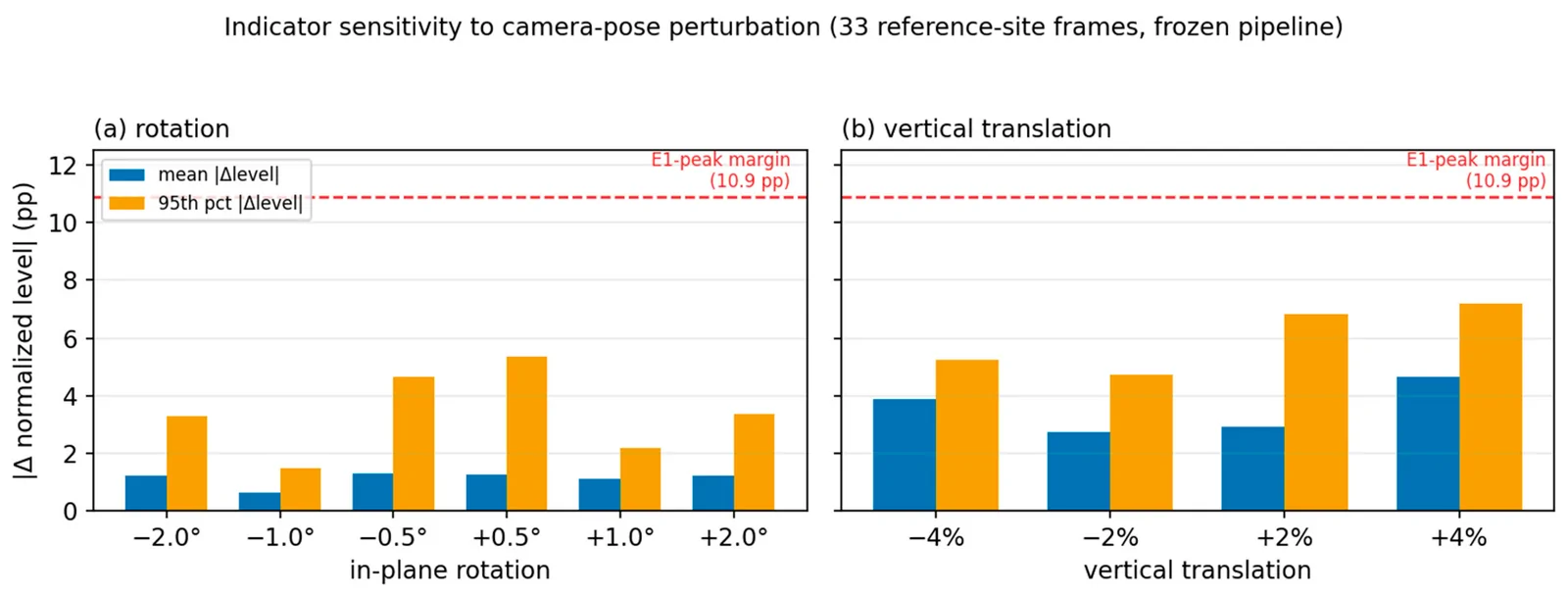
Sensitivity of the normalized level indicator to camera-pose perturbation on the 33 reference-site verification frames: (**a**) in-plane rotation; (**b**) vertical translation. Bars: mean |Δlevel|; whiskers: 95th percentile. Dashed line: ≈11 pp margin between the E1 event peak (72.9%) and the control-band edge (62%).

(a) Model confidence threshold and prompt. Recomputing the frozen pipeline on the 79-frame verification subset with confidence thresholds 0.15–0.35 moves the water-positive mIoU smoothly from 0.653 (0.15) through 0.633 at the reported operating point 0.25 down to 0.614 (0.35)—a total span of 0.04 with no abrupt transition—and the per-quadrant mIoU values vary by less than 0.04 across the same range (Figure 10a). The reported threshold therefore sits on a flat region of the response surface rather than at a fragile tuned optimum. Prompt wording matters more: at confidence 0.25, “water surface” scores 0.483, “standing water” 0.591, “water” 0.633, and “flooded water” 0.663—but the last simultaneously inflates the negative-frame false-positive area from 0.15% to 3.25%, a 22-fold increase (Figure 10b) that disqualifies it for alarm use. The single-token prompt “water” offers the best accuracy–specificity compromise, corroborating the qualitative observation of Section 4.1 that single-token prompts suffice for this scene.
(b) Alarm-rule parameters. Re-running both alarm rules over a 45-point grid—level threshold θ ∈ {60, 61, 62, 63, 64} × water-area bound {45, 50, 55} × 5-min rise bound {2, 3, 4} percentage points—shows that the qualitative conclusions are parameter-independent: the original five negative days produce at most one alarm frame in any setting (0–1/102), the prospective dry days produce alarms on 1–6 of 13 days, and the 11 August event detections are preserved at all 45 settings (8–50 event alarm frames, depending on strictness). The choice θ = 62% is thus not an artifact of fine-tuning against the negative days.
(c) Camera-pose perturbation. On the 33 reference-site verification frames we perturbed the input images by ±0.5°, ±1°, and ±2° in-plane rotations and ±2% and ±4% vertical translations before re-running the frozen pipeline (Figure 11). Rotations shift the level indicator by a mean |Δ| of 0.65–1.28 pp (95th percentile 1.5–5.3 pp)—small relative to the ≈11 pp separation between the event peak (72.9%) and the upper edge of the control-day band (62%)—whereas translations are more influential (mean |Δ| 2.7–4.7 pp) yet still below the alarm margin. In 8 of the 33 frames the perturbation tipped an already-borderline dusk frame into detection failure—the same mechanism as the failure modes of Section 5.6—producing the large (up to ≈22 pp) tail values; these frames already sat near the validity boundary, so the tail characterizes marginal frames rather than typical behavior. Small pose drift therefore does not threaten event detection at this site, but it motivates pose-invariant indicators and periodic pose re-normalization as hardening steps (Section 6).
(d) Annotation tolerance. Morphologically dilating and eroding the 79 expert reference masks by 1–5 px and re-scoring the unchanged predictions moves the macro-averaged mIoU by at most 0.003 (0.632–0.633), and perturbed and original references agree at IoU ≥ 0.95. The reported pixel-level differences between quadrants and between methods are therefore an order of magnitude larger than the boundary-convention uncertainty of the reference masks themselves.
(e) Inter-annotator agreement. To quantify human-label variability directly, a second independent annotator re-labeled all 79 frames under the identical polygon protocol (Section 3.2 (iv)), with the annotation convention documented and the first annotator’s masks available only as a semi-transparent overlay that the second annotator was instructed to judge independently rather than trace. On the 64 water-positive frames the two annotators agree at mean IoU 0.706 (95% bootstrap CI 0.649–0.758) and Dice 0.800; on the 15 water-free frames they agree on emptiness for 13. The disagreement is not uniform: four frames—all morning scenes with only thin residual water films (RP-001, RP-003, UP-001)—show categorical disagreement (one annotator traces a specular sheet, the other judges the surface merely wet), and excluding this thin-film regime raises agreement to IoU 0.753 (Dice 0.852) on the remaining 60 frames. Two readings follow. First, the reference masks are reproducible: annotator disagreement is confined to exactly the wet-vs-ponded boundary cases that Section 5.7 already flags, not to ordinary open-water extent. Second, the zero-shot model’s 0.633 mIoU against the first annotator sits only ≈0.07 below the 0.706 human–human agreement on the same 64 frames—i.e., a substantial fraction of the model’s residual error is the irreducible annotation-convention variance measured here rather than segmentation error. The agreement is also stage- and scene-stratified in an operationally favorable way. Split by the reference waterline level at the canal site (where the 62% alarm line applies), inter-annotator IoU rises with water stage—0.746 below the line (*n* = 24) versus 0.811 at or above it (*n* = 8), and 0.717/0.768/0.803 across low/middle/high terciles—so the disagreement-prone regime is the low-water one, whose misreads carry no consequence for drainage safety, while the safety-critical near-warning regime is where human agreement is highest. Split by scene category, road-ponding frames score lowest (0.603, *n* = 11), consistent with the mixed submerged-object convention (vehicles, pillars) being the dominant human disagreement there, whereas pump-station aprons score highest (0.822); across all positive frames, small water extents agree worse than large ones (0.623 vs. 0.790 across the median split), again localizing annotation variance to thin, boundary-dominated water rather than to open-water extent.

### 5.10. Runtime and Deployment Cost

Zero-shot inference costs ≈ 46 ms per frame at 644 × 644 on an RTX 4060 Laptop GPU (8 GB, FP16, batch 1), i.e., ≈22 FPS against capture intervals of 300–3600 s, so multi-site processing on a single consumer GPU is plausible, though end-to-end multi-camera load testing remains future work. No task-specific training or annotation was performed for the monitoring pipeline itself; manual annotation was required only for the 79-frame evaluation subset. By contrast, supervised alternatives require pixel-level labeling—reported at ≈1 h per image in the FloodNet documentation [9]—and multi-GPU training budgets [6,21,22,23,24]. For continuous edge deployment, the foundation model can either run on station-level GPUs or serve as an annotation engine to distill lightweight students (e.g., PP-LiteSeg, DDRNet) [22,23] for NPU-class hardware [25,26,27]. The pixel-level verification above suggests a practical division of labor: zero-shot masks are already reliable enough to propose labels [31], with expert review focused on the boundary band and the identified failure regimes.

## 6. Discussion

What zero-shot buys. The results reframe the build-versus-reuse question for drainage monitoring: for fixed-camera water-extent monitoring, a text-prompted foundation model already delivers indicators useful for operational evaluation—both heavy-rain events of 10 August and the 7 July evening storm were detected by the compound rule, with no alarms on the 102 examined negative-day frames in the retrospective evaluation and, prospectively, only 6 alarm frames out of 784 across 13 verified dry days (0.77%—all single-frame illumination-switchover transients) plus correct detection of a newly encountered heavy-rain day that had never been used for threshold or rule design—without any labeling or training. Pixel-level verification on 79 frames from 24 sites (mIoU 0.633, 0.652 excluding failure frames; 0.15% mean false-positive area on 15 water-free frames) shows these indicators rest on segmentation that is accurate where it matters and conservative where it is uncertain, and this reading is stable: the mIoU varies by less than 0.04 across confidence thresholds 0.15–0.35 at the reported prompt, and the four prompt wordings span 0.483–0.663 with “water” the best accuracy–specificity compromise (Section 5.9 (a)). Most tellingly, the model’s 0.633 mIoU approaches the 0.706 inter-annotator IoU that two independent human annotators achieve on the same frames (Section 5.9 (e)): the residual gap to perfect agreement is largely label-convention variance rather than segmentation error. The baseline comparison (Section 5.8) sharpens the point: an open-vocabulary detector with the same prompt misses most of the water (recall 0.302) and scores zero mean IoU on night/no-rain infrared frames, while a classical color rule achieves recall only by flooding the frame with false positives (68% of pixels on water-free frames)—segmentation error profiles that may limit their suitability for the proposed alarm logic, which would require direct end-to-end evaluation. This is particularly valuable for drainage networks with hundreds of heterogeneous camera poses, where per-site supervised training is impractical [32].

Where it breaks. The failure taxonomy (Section 5.6 and Section 5.7) is specific and bounded: heavy-rain obscuration, sediment-turbid low-light water, vegetation-covered water, and—revealed by the expanded 79-frame verification—thin residual water films on morning road surfaces in the reference frames, which the model systematically under-reports; the prospective validation adds a fifth, operational mode—single-frame segmentation transients at the dawn infrared-to-color illumination switchover, which are imaging artifacts rather than hydrological signals. The first two are transient and detectable as anomalies (empty or implausibly small masks in an always-wet scene), so a production system can flag low-confidence frames rather than silently failing. Offline or delayed temporal fusion (e.g., majority voting over ±2 frames—at 5-min sampling, a ≈10-min confirmation delay) could mask most single-frame failures; indeed, the two-frame persistence rule evaluated post hoc in Section 5.4 removed all six prospective false alarms while preserving every real detection, which we take as evidence that temporal hardening targets the right failure mode. We report per-frame results deliberately to expose the raw model behavior. The thin-film weakness primarily affects quantification on morning road scenes rather than event detection, since such films persist after the event peak has passed; where residual-film mapping matters, a lower confidence threshold or a site-adapted prompt (“wet road”) is a straightforward remedy. Vegetation-covered water, by contrast, is a persistent site property and calls for a site-specific prompt set or light fine-tuning rather than temporal smoothing.

Negatives and confounders matter. The 4 September false alarms trace to a camera re-aiming between audit days—an operational event that event-only evaluation would never reveal, and whose image-space indicator signature (a persistent offset in the sampled post-change frames) is indistinguishable from pump-station regulation without inspecting the imagery itself. We echo and quantify, for the zero-shot setting, the concern raised by Aunet [6] about missing negative samples: alarm logic must be validated on negative days that include operational dynamics, not only on rainy imagery. That the compound rule absorbed a permanent pose shift without retraining suggests indicator designs based on relative temporal change and cross-feature agreement are more deployment-robust than absolute image-space thresholds; pose-invariant indicators (e.g., waterline expressed relative to detected bank landmarks) are a natural hardening step.

From normalized to metric levels. Our waterline indicator is normalized (percentage of frame height). Mapping it to metric water depth requires per-site geometric calibration, e.g., homography-based virtual staff gauges [7] or the visible depth ruler present at one underpass site (Figure 1f); dedicated calibrated AI gauges report MAEs around 1–3 cm [2]. Conceptually, this calibration is a one-time per-site registration step rather than a model change: given a structure of known physical size in the field of view, its pixel extent *h_pix_* defines the image scale, and a waterline displacement of Δ*y* pixels then maps to a metric depth change on the order of *k*·Δ*y*·cos(*tilt*)/*h_pix_* (with *k* a unit constant and *tilt* the camera’s inclination), or equivalently through a single homography for planar water surfaces [7]. Combining zero-shot segmentation with such geometric calibration would enable direct evaluation against physical water-level measurements, and is the natural next step toward metric alarm thresholds.

Limitations. (i) Multi-day coverage is currently available for one reference site; the other 24 sites are represented on a single event day. (ii) Ground truth for rain windows is visual/operator-verified rather than gauge-measured rainfall; co-located rain-gauge telemetry would strengthen event timing. (iii) The 79-frame pixel-level subset, while stratified across quadrants, sites (24 of 25), and failure modes, remains modest relative to the full benchmark, and its reference masks are expert polygons whose boundary convention contributes a few points of irreducible IoU uncertainty—though an annotation-tolerance analysis bounds this effect to ≤0.003 mIoU under ±1–5-pixel mask perturbations (Section 5.9 (d)), and a second independent annotator re-labeled all 79 frames, yielding inter-annotator IoU 0.706 and Dice 0.800 on the water-positive frames with disagreement concentrated in the thin-film regime (Section 5.9 (e)). A larger, pixel-fine, multi-annotator study remains future work. (iv) All sites are in one city; cross-city generalization remains untested. (v) Baselines were evaluated at their training-free operating points; supervised per-site fine-tuning of the baseline families would likely raise their scores at the annotation cost our pipeline is designed to avoid, and we did not benchmark detector–segmenter cascades such as Grounded-SAM-style open-vocabulary grounding pipelines, which we defer to future work. (vi) The alarm rules and threshold were designed on the original negative-day data; we addressed this by prospective validation on 14 additional days never used for design, where the frozen rule produced 0.77% alarm frames on 13 verified dry days and detected a new event day—still, only two additional event days have been evaluated, and the two-frame persistence variant of Section 5.4 is post hoc with respect to those days, so a fully prospective re-validation of the hardened rule is required before deployment claims. (vii) Frames within a day are temporally correlated, so frame-level rates overstate effective sample sizes; we therefore report day-level false-alarm counts with Clopper–Pearson bounds where prospective days allow (Section 5.4). (viii) The annotation target mixes visible water with occluded-but-submerged ground (amodal convention), the masks are single-annotator work, and no co-located physical water-level measurements were available as absolute ground truth. (ix) The prospective campaign revealed a new failure mode—single-frame segmentation transients at illumination-mode switchover—that the recommended persistence rule mitigates only a posteriori; whether persistence holds across seasons and camera models requires the prospective re-validation noted in (vi).

## 7. Conclusions

This paper presented a quadrant-wise (day/night × rain/no-rain) benchmark of zero-shot water segmentation on fixed drainage surveillance imagery: 3651 frames from 25 cameras across four scene categories, including two heavy-rain-event days and five verified negative days. Text-prompted SAM 3 segmented water successfully in 96.9% of diurnal-cycle frames, detected both heavy-rain events of 10 August 2026 (peaks 72.9% and 71.4% against a 43–62% control band) with city-wide cross-site corroboration, and—under a compound area/rise-rate alarm rule—produced no alarms on the 102 examined negative-day frames, versus 12/102 for a level-only rule exposed to a real camera re-aiming event (a retrospective, in-sample comparison). Pixel-level verification on 79 expert-annotated frames from 24 sites confirms the operational reading of these indicators: mean water-class IoU 0.633 across the 64 water-positive frames (0.652 when excluding two failure frames, as a sensitivity analysis), night precision above 0.92, and near-zero false positives (mean 0.15% of pixels) on 15 water-free frames. On identical reference masks, zero-shot SAM 3 clearly outperforms an open-vocabulary detector baseline (YOLOE, mIoU 0.268, zero mean IoU on night/no-rain infrared frames) and a classical HSV color rule (mIoU 0.453, with a 68% false-positive area on negatives that precludes alarm use). Failures are specific and reproducible under the sampled conditions (heavy-rain obscuration, sediment-turbid dusk water, vegetation-covered water, thin morning water films, illumination-switchover transients), delineating the operating envelope. These findings are robust rather than tuned: the mIoU varies by less than 0.04 across confidence thresholds 0.15–0.35 and by 0.18 across four prompt wordings (with “water” the best accuracy–specificity compromise), the alarm-rule conclusions hold across a 45-point parameter grid, small camera-pose perturbations (±0.5–2° rotation) shift the level indicator by ≈1 pp on average against an ≈11 pp alarm margin, and ±1–5-pixel reference-mask perturbations move the mIoU by at most 0.003, while a second independent annotator reproduced the reference masks at inter-annotator IoU 0.706 (Dice 0.800)—the human agreement level that the zero-shot 0.633 approaches. Prospectively, the frozen pipeline and rules produced 0.77% alarm frames on 784 frames across 13 verified dry days—all single-frame illumination-switchover transients that a two-frame persistence rule removes while preserving every real detection—and correctly detected a newly encountered heavy-rain day never used for threshold or rule design. At ≈46 ms per frame on a consumer laptop GPU (not an edge platform) with zero annotation, zero-shot foundation models are a promising candidate for operational evaluation on drainage surveillance networks and, in the nearer term, as annotation engines for distilling edge-deployable students. Future work includes metric level calibration via virtual staff gauges, pose-invariant indicator design, a fully prospective re-validation of the persistence-hardened alarm rule, supervised and Grounded-SAM-class baselines, site-adapted prompts for vegetation-covered water, and extension to the full multi-day, multi-city archive.

## Figures and Tables

**Table 1:** Composition of the UWS-City benchmark. Site identifiers are anonymized (scene group + number); the mapping is held privately by the authors.

Scene Category	Site IDs	Sites	Frames	Days Covered	Role
River/canal	RV-001–RV-006	6	963	10 August (all); 7 July + 5 negative days (RV-001)	Event day + reference site
Road ponding	RP-001–RP-007	7	905	10 August	Event day, multi-site
Underpass	UP-001–UP-006	6	889	10 August	Event day, multi-site
Pump-station apron	PS-001–PS-006	6	894	10 August	Event day, multi-site
Total	—	25	3651	7 days	—

**Table 2:** Quadrant-wise zero-shot segmentation statistics (reference site RV-001, 10 August 2026, 162 frames).

Quadrant	Frames	Detection Success (%)	Mean Area (%)	Mean Level (%)	Max Level (%)
Day/No-rain	52	100.0	46.5 ± 3.4	58.8	66.3
Day/Rain	44	97.7	51.5 ± 10.9	61.4	72.9
Night/No-rain	30	100.0	42.0 ± 6.6	56.1	62.9
Night/Rain	36	88.9	49.7 ± 17.9	65.2	71.4
Overall	162	96.9	—	—	—

**Table 3:** False-alarm analysis on negative days (threshold θ = 62%).

Day	Frames	Alarms, Rule A (Level-Only)	Alarms, Rule B (Compound)	Max Level (%)
18 August 2026	17	0	0	59.9
19 August 2026	18	0	0	58.2
20 August 2026	24	0	0	56.6
26 August 2026	21	0	0	53.0
9 September 2026	22	12	0	62.9
Total	102	12 (11.8%)	0 (0.0%)	—

**Table 4:** Pixel-level verification of zero-shot masks against expert reference polygons (79 frames, 24 sites). IoU, precision, and recall are water-class metrics macro-averaged over the 64 water-positive frames; the negative row reports the false-positive pixel rate only.

Subset	*n*	Mean IoU	Mean Precision	Mean Recall
Day/no rain	18	0.533	0.673	0.632
Day/rain	17	0.690	0.836	0.754
Night/no rain	10	0.660	0.925	0.704
Night/rain	19	0.662	0.931	0.692
All water-positive	64	0.633	0.832	0.693
All positive, excl. 2 failure frames (sensitivity)	62	0.652	—	—
Negative frames (water-free)	15	—	—	—

Negative frames: mean false-positive pixel rate 0.15% (max 2.22%). Inter-annotator agreement (second independent annotator vs. reference): mean IoU 0.706, Dice 0.800 on the 64 water-positive frames; 13 of 15 water-free frames agreed empty (Section 5.9 (e)).

**Table 5:** Baseline comparison on the 79-frame reference set (64 water-positive frames; identical prompt “water” for SAM 3 and YOLOE).

Method	Type	Mean IoU	Mean Precision	Mean Recall	Negative-Frame FP Area, Mean (Max)
SAM 3 text prompt (this work)	foundation model, zero-shot	0.633	0.832	0.693	0.15% (2.22%)
YOLOE-v8s-seg, text “water”	open-vocabulary detector	0.268	0.341	0.302	0.00% (0.00%)
HSV threshold	classical color rule	0.453	0.546	0.730	68.17% (90.46%)

## Data Availability

The raw surveillance frames are not publicly available because they originate from an operational urban drainage network whose camera identifiers, network topology, and storage endpoints are subject to infrastructure-security restrictions; all identifiers are anonymized in this paper. To support reproducibility, we will release upon acceptance the anonymized 79-frame verification subset, the expert and second-annotator reference masks, the zero-shot predictions, the per-frame derived indicators (water-area ratio, normalized waterline level) for all benchmark days, the alarm-rule implementation and evaluation scripts, and the exact model checkpoint identifier and inference configuration. Raw network imagery remains available from the corresponding author on reasonable request, subject to a data-use agreement with the network operator.
